# Account of Diseases in an Eastern District of London, from August 20 to Sept. 20, 1804

**Published:** 1804-10-01

**Authors:** 


### *Account of Diseases in an Eastern District of London, from August 20 to Sept. 20, 1804.*

| | |
|---|---|
| **ACUTE DISEASES.** | |
| Typhus | 3 |
| Pneumonia | 2 |
| Dysenteria | 3 |
| Cholera | 4 |
| Rheumatismus Acutus | 7 |
| **CHRONIC DISEASES.** | |
| Tussis | 9 |
| Dyspnœa | 5 |
| Tussis cum Dyspnœa | 10 |
| Pleurodyne | 2 |
| Phthisis Pulmonalis | 3 |
| Hydrothorax | 5 |
| Ascites | 4 |
| Hysteria | 4 |
| Chlorosis | 5 |
| Diarrhœa | 15 |
| Colica | 3 |
| Gastrodynia | 10 |
| Cephalalgia | 5 |
| Herpes | 2 |
| Prolapsus Vaginæ | 1 |
| Menorrhagia | 4 |
| Ischuria | 1 |
| Vermes | 3 |
| Podagra Atonica | 1 |
| Rheumatismus Chronicus | 15 |
| **PUERPERAL DISEASES.** | |
| Ephemera | 7 |
| Menorrhagia Lochialis | 8 |
| Mastrodynia | 4 |
| **INFANTILE DISEASES.** | |
| Tabes Mesenterica | 1 |
| Aphthæ | 4 |
| Vermes | 2 |
| Diarrhœa | 6 |
| Dentition | 1 |

